# Testosterone-Induced Expression of Male Colour Morphs in Females of the Polymorphic Tawny Dragon Lizard, *Ctenophorus decresii*

**DOI:** 10.1371/journal.pone.0140458

**Published:** 2015-10-20

**Authors:** Katrina Rankin, Devi Stuart-Fox

**Affiliations:** School of BioSciences, The University of Melbourne, Parkville, Victoria, Australia; University of Lausanne, SWITZERLAND

## Abstract

Many colour polymorphisms are present only in one sex, usually males, but proximate mechanisms controlling the expression of sex-limited colour polymorphisms have received little attention. Here, we test the hypothesis that artificial elevation of testosterone in females of the colour polymorphic tawny dragon lizard, *Ctenophorus decresii*, can induce them to express the same colour morphs, in similar frequencies, to those found in males. Male *C*. *decresii*, express four discrete throat colour morphs (orange, yellow, grey and an orange central patch surrounded by yellow). We used silastic implants to experimentally elevate testosterone levels in mature females to induce colour expression. Testosterone elevation resulted in a substantial increase in the proportion and intensity of orange but not yellow colouration, which was present in a subset of females prior to treatment. Consequently, females exhibited the same set of colour morphs as males, and we confirmed that these morphs are objectively classifiable, by using digital image analyses and spectral reflectance measurements, and occur in similar frequencies as in males. These results indicate that the influence of testosterone differs for different colours, suggesting that their expression may be governed by different proximate hormonal mechanisms. Thus, caution must be exercised when using artificial testosterone manipulation to induce female expression of sex-limited colour polymorphisms. Nevertheless, the ability to express sex-limited colours (in this case orange) to reveal the same, objectively classifiable morphs in similar frequencies to males suggests autosomal rather than sex-linked inheritance, and can facilitate further research on the genetic basis of colour polymorphism, including estimating heritability and selection on colour morphs from pedigree data.

## Introduction

The study of colour polymorphism, that is, the presence of multiple, discrete, genetically determined colour forms within one sex and within an interbreeding population [1], has provided fundamental insights into evolutionary processes and phenomena such as frequency dependent selection, speciation, mimicry and sexual selection [2–8]. Many colour polymorphisms are present only in one sex (sex-limited), usually males, presumably because the polymorphic colour signal is sexually selected. Indeed many polymorphic male colour signals have a demonstrated function in male-male competition, female mate choice or both, and are associated with alternative reproductive or life-history strategies [2, 7, 9, 10]. Despite the prevalence of colour polymorphic species as model study systems in evolutionary biology, proximate mechanisms controlling the expression of colour polymorphisms have been identified in relatively few species [11, 12–24].

The sex steroid testosterone is often critical for the expression of sexually dimorphic colour signals in males [13, 24–26]. One approach to qualifying the effect of testosterone on colour expression is to test whether it induces colour expression in females that lack the male colour signal. For example, artificial elevation of testosterone in females induces expression of the male colour morphs in colour polymorphic birds [21] and lizards [14]. Testing whether artificial elevation of testosterone induces sex-limited colour polymorphism in females provides important insight into the degree to which testosterone has an activational influence on colour expression and whether this differs for different colours. Furthermore, inducing the expression of the polymorphism in females is useful to infer underlying patterns of inheritance (e.g. autosomal or sex-linked) or heritability of colour signals, which can be estimated from pedigrees with more confidence when females phenotypically express the underlying genotype [21]. To reliably score the underlying genotype, however, requires that testosterone consistently induces females to express the same set of discrete colour morphs that are present in males, in similar frequencies. Therefore, it is important to quantify changes in female expression of each colour component in response to testosterone and compare objective classification of observed colour morphs in males and females.

Here, we artificially elevate testosterone in female tawny dragon lizards, *Ctenophorus decresii*, to test whether testosterone induces expression of the male colour polymorphism. *Ctenophorus decresii* is a small, sexually dimorphic agamid lizard (mean snout-vent length 80 mm and 70 mm for males and females, respectively), in which males develop and express four discrete throat colour morphs at sexual maturity: grey, orange, yellow and orange+yellow (an orange central patch surrounded by yellow) [27]. Females have cream throats, sometimes with flushes of yellow. In this study, we used silastic implants to experimentally elevate testosterone levels in mature females to within the normal physiological range of free-ranging males. To assess the effect of testosterone on colour expression and how this may differ between orange, yellow and grey throat colour components, we objectively quantified the change in the quality and extent of colouration over time using calibrated digital photographs and spectrophotometry. We tested whether females express the same discrete morphs in similar frequencies to males to test whether experimental testosterone manipulation can be used to reliably score the phenotypic expression of the underlying genotype.

## Methods and Materials

### Animal capture and husbandry

Trials were performed using a captive population of 35 female *C*. *decresii*, comprising 16 mature individuals originating from Warren Gorge, Flinders Ranges National Park, South Australia (31.4222° S, 138.7050° E), captured in October and November 2011, and 19 of their offspring bred in captivity.

Lizards were transferred to the animal facility at the University of Melbourne where they were housed individually in 55 L x 34 W x 38 D cm opaque plastic tubs containing a layer of sand, and provided with a ceramic or terracotta tile hide. The room was maintained at temperatures and lighting regimes that mimicked natural seasonal variation. A heat lamp was suspended in each enclosure to generate a thermal gradient, and allow animals to attain their preferred body temperatures (approx. 36°C; Walker unpublished data). Lizards were misted and fed live crickets three times per week.

### Ethics statement

This study was approved by The University of Melbourne Animal Ethics Committee (approval 1011760), and animals collected and transported under a Department of Environment and Primary Industries (DEPI) Scientific permit (10005980), DEPI Import permits (14087874, 14078381), Department of Environment and Natural Resources (DENR) Export permits (E22641, E22520), DENR Collection permit (E25861), and South Australian DENR Wildlife Ethics permits (18/2010, 18/2010-M1 and 35/2013).

### Testosterone implantation

Prior to implanting, lizards were sterilised dorsally by wiping with 70% ethanol and injected subcutaneously with 0.2 mL lidocaine (lignocaine hydrochloride 2%; Cenvet, Kings Park, NSW, Australia). The lizards were then cooled on ice to induce immobility. Sterile silastic implants (4 mm lengths, 1.47 mm internal diameter, 1.96 mm external diameter; Dow Corning, Midland, MI, USA) packed with crystalline testosterone powder (no. T1500, Sigma), and closed with 1 mm silastic adhesive at either end were inserted through a small dorsolateral incision near the shoulder. The incision was then closed with medical adhesive silicone (Dow Corning, Midland, MI, USA).

### Testosterone assays

Blood samples (~100 μL) were taken from each female at three time points (10 days prior to implantation, hereafter “pre- T”, and 1- and 4- weeks post implantation) by venipuncture from the vena angularis (in the corner of the mouth). Plasma was harvested from whole blood by centrifugation and frozen at -20°C until assayed.

Plasma levels of testosterone were measured using an enzyme immunoassay kit (Cayman Chemical, Ann Arbor, MI USA). Testosterone assay plates, with standards and samples (in duplicate), were prepared to manufacturer's instructions and absorbance was measured at 405 nm on a plate reader (BioTek ELx800UV; BioTek Instruments, Winooski, VT USA) using Gen5 v.2.05 software (BioTek Instruments, VT USA).

Testosterone assay plates, with standards and samples (in duplicate), were prepared to manufacturer's instructions and absorbance was measured at 405 nm on a plate reader (BioTek ELx800UV; BioTek Instruments, Winooski, VT USA) using Gen5 v.2.05 software (BioTek Instruments, VT USA).

### Quantifying colour development

Colour development was monitored through weekly photographs taken under standardised light conditions and spectral reflectance measurements of each lizard for twelve weeks. For photographs, we used a Canon PowerShot SX1-IS digital camera (saved in RAW format). We calibrated the images with respect to radiance and light intensity [28]. This entailed first applying a function to linearize camera responses (R, G, B and luminance values) in relation to six grey-scale squares of a colour checker standard (X-Rite Inc., Grand Rapids, MI, USA) and their measured reflectance values [28]. Next, we equalised RGB values relative to a grey photographic standard included in each photo (30% reflectance) to remove any effect of minor variation in lighting [28]. We then performed a segmentation analysis on calibrated photos to quantify the proportion of yellow, red and grey on the throat of each individual, as described in Teasdale, Stevens [27]. This analysis standardised for luminance, and extracted portions of the cropped throat area based on the RGB values of each pixel according to set threshold values. The thresholds we used to determine the colour layers in this analysis were 0.20 for red and 0.15 for yellow, assigned based on an initial analysis of a subset of the images. We set a conservative (higher) threshold for red to ensure that orange and yellow throat colouration could be clearly distinguished. Although photos only capture information on colour in the human-visible spectrum (400–700 nm), *C*. *decresii* throat colours have minimal ultraviolet reflectance (300–400 nm; S1 Fig). Image calibration and segmentation analyses were done using modified scripts written by John Endler and Martin Stevens, executed in MATLAB (The MathWorks, Inc., MA, USA).

We quantified the spectral properties of female throat colouration at one-week intervals following implantation. We took reflectance measurements using an Ocean Optics USB2000 + spectrometer and PX-3 Pulsed Xenon light source, both connected to a probe via a bifurcated fibre-optic cable. Measurements were of an oval point sample 3 mm x 4 mm, and taken relative to a 99% diffuse white reflectance standard in the range of 300–700 nm, the visual spectrum for most lizards [29]. Measurements were taken of the throat region and bib (coloured region under the chin; a fold of skin between the throat proper and the chest [30, 31]) at a 45° angle to the surface of the lizard. We derived measures of the achromatic (luminance) and chromatic (colour) contrast of the primary throat colour of each individual against a neutral background (30% grey) to the likely visual system of *C*. *decresii*. Briefly, we applied a model of colour discrimination, which assumes that discrimination is limited by photoreceptor noise [32, 33]. The model estimates the ‘distance’ between two colours (in this case throat and neutral grey background) in units of just noticeable differences (JND), where one JND is the threshold of discrimination and larger numbers correspond to increasingly different colours. For this model, we assumed an irradiance spectrum of full sunlight, a uniform 30% reflectance grey background and photoreceptor spectral sensitivities of UVS λ_max_ = 365 nm, SWS λ_max_ = 440 nm, MWS λ_max_ = 493 nm, LWS λ_max_ = 571 nm. These values are those for the closely related ornate dragon, *Ctenophorus ornatus* [34], with the addition of a UVS cone, which was not identified in the microspectrophotometry (MSP) study of Barbour, Archer [34], likely due to the difficulty of isolating individual photoreceptors in lizard retinas using MSP methods ([34], Yewers et al. in press). These assumptions are validated by our recent analyses of the *C*. *decresii* visual system [Yewers et al. in press], which confirms photoreceptor spectral sensitivity λ_max_ values within two to four nm for the SWS, MWS and LWS cones and the retinal expression of a UVS opsin gene as found in all other diurnal lizards [35]. We assumed that photoreceptor noise (ω_i_) for the LWS photoreceptor = 0.05 and then derived ω_i_ for remaining photoreceptor classes [33, 36]. We used a ratio of 1: 1: 3.5: 6 for the four single receptor classes based on the relative photoreceptor frequencies in Barbour, Archer [34]. Full model calculations are detailed in Stuart-Fox, Moussalli [36] and Teasdale, Stevens [27].

### Statistical analysis

To quantify the change in circulating testosterone in implanted females, we performed a one-way ANOVA (PROC GLM, SAS 9.2) on a subset of 18 females for which we had successfully taken repeat blood samples. Plasma samples were collected from blood taken pre-T implantation (n = 18), one week post-implantation (n = 11), and four weeks post-implantation (n = 15). Testosterone concentration (ng/mL) was compared across all three time points within females, and assessed relative to free-ranging males.

We visually assigned each female to a morph category based on its peak colour expression (weeks 3 to 12, depending on the individual). To check whether our visually assigned morphs could be objectively classified based on seven colour variables (proportion red, yellow and grey, and chromatic and achromatic contrast of the primary throat colour and bib), we conducted a discriminant function analysis (DFA; SAS 9.2) with morph category as the classification variable. This analysis generates a linear combination of the canonical variables that maximizes the probability of correctly assigning observations to their predetermined groups (in this case, the four colour morphs). Comparison of the frequency of each colour morph by sex was analysed using Fishers exact test, given the low sample size (STATA 13.1).

To analyse the colour development over time, we used a general linear mixed model (GLMM; SAS 9.2), with each of the colour variables as the dependent variables, and time (weeks 1–6, and 10 and 12), visually assigned colour morph and their interaction as the independent variables (fixed factors). The inclusion of ‘colour morph’ as a fixed factor tested how the change in colour expression over time differed for different morphs. We tested for both linear and quadratic relationships between colour variables and time, as well as the interaction between quadratic time effects and colour morph. Female ID was included as a random factor to account for repeated measures on individual females. For significant effects, we applied false discovery rate corrections [37] to assess pairwise differences between morphs and between weeks.

## Results

**Fig 1:**
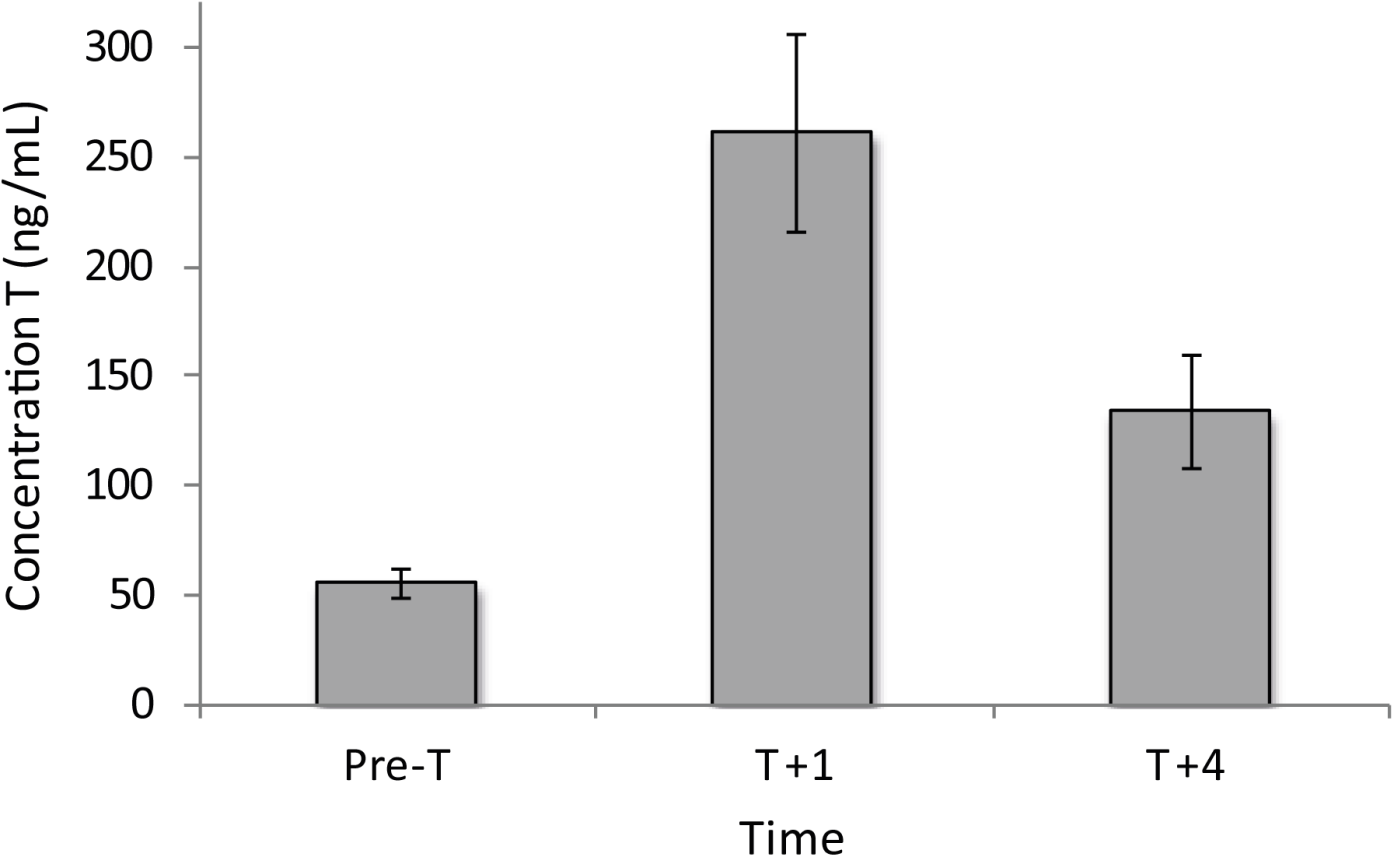
Effects of experimental elevation of testosterone. Change in mean circulating testosterone (T) concentrations in female *C*. *decresii* pre- implant, 1 week post-implant, and 4 weeks post-implant.

**Fig 2:**
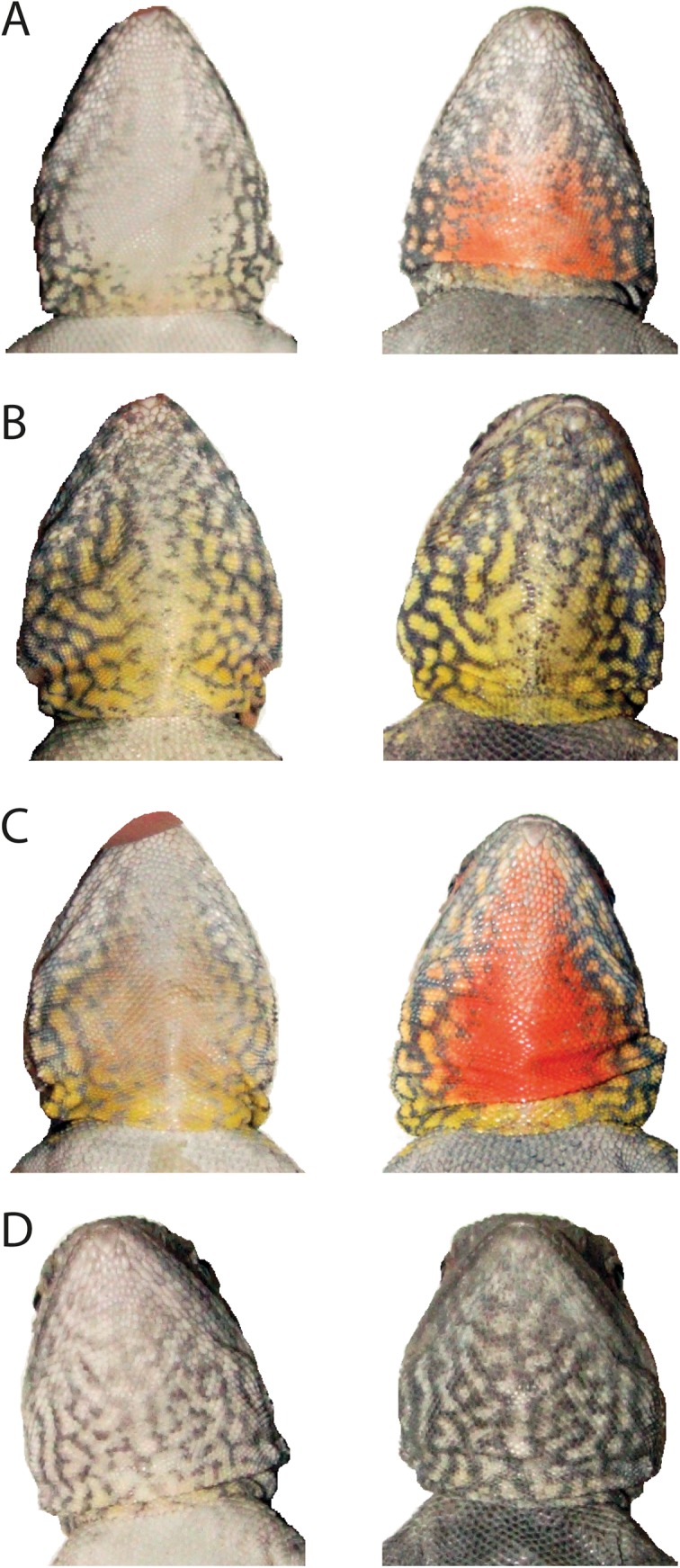
Effect of testosterone on female throat colour expression for each morph. Pre-testosterone implantation (left) versus same lizard at peak of morph expression (right): **A)** orange, **B)** yellow, **C)** orange+yellow, **D)** grey.

Implantation via silastic implants significantly increased circulating testosterone of female *C*. *decresii* through time (F_2, 24_ = 22.01, p < 0.0001). Testosterone concentration in blood plasma increased from 55.95 ± 6.4 ng/mL pre-T to 262.16 ± 45.09 ng/mL at one-week post implantation (pre-implantation versus week 1: t_24_ = -6.63, p < 0.0001). This then declined to 134.02 ± 25.47 ng/mL at four weeks post-implantation (pre-implantation versus week 4: t_24_ = -2.65, p = 0.036, week 1 versus week 4: t_24_ = 4.03, p = 0.0014) (Fig 1). These values are biologically relevant, as they are within the normal physiological range of wild caught males of the same species (mean ± SE = 62.38 ± 7.32 ng/mL; range = 3.69 to 353.59 ng/mL; n = 92; Yewers and Stuart-Fox, unpublished data). This increase in testosterone induced females to express throat colour (Fig 2). After testosterone-induced colour expression, females were visually assigned to one of the four male colour morph categories, with ten females assigned to orange, seven to yellow, seven to grey and eleven to orange+yellow morphs. There was no significant difference in the frequency of colour morphs between males and females (Fishers exact p = 0.98).

**Fig 3:**
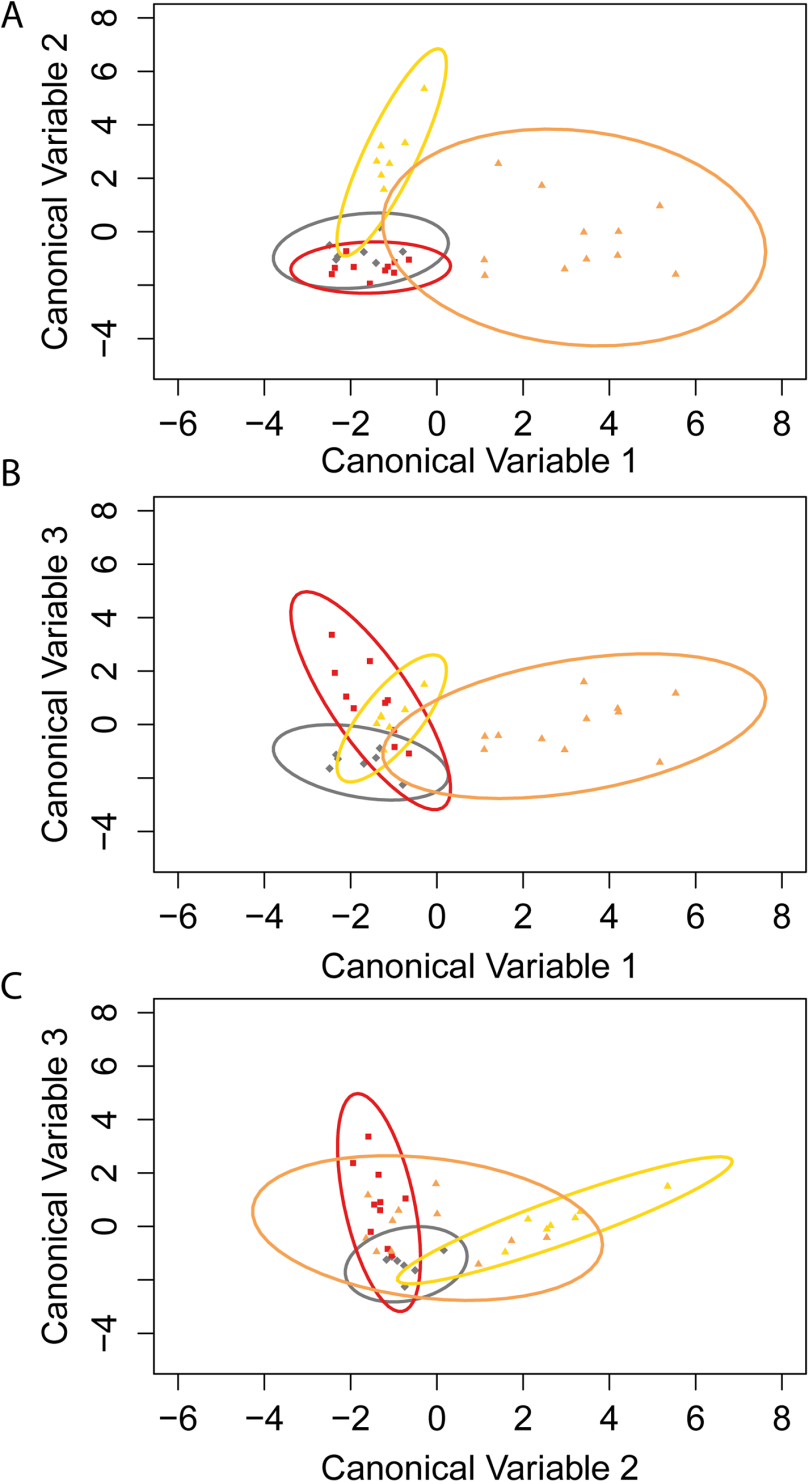
Discriminant function analysis (DFA) confirming the presence of four discrete throat colour morphs following testosterone implantation. Orange: red squares; orange+yellow: orange triangles; yellow: yellow triangles; and grey: grey diamonds. Individuals are plotted for canonical variables **A)** 1 and 2; **B)** 1 and 3; and **C)** 2 and 3, with 95% confidence ellipses.

The visually assigned morphs were confirmed by a discriminant function analysis (DFA). Discrimination of the morphs was highly significant (Wilks’ λ = 0.02, F_21, 73_ = 9.02, p < 0.0001; Fig 3), and canonical variables 1 and 2 explained 60.8% and 30.9% of the variation in female throat colouration, respectively. The remaining 8.28% was explained by canonical variable 3. Canonical variable 1 most clearly differentiated orange+yellow morphs, with high proportions of red and yellow, a low proportion of grey, and low achromatic contrast of the throat and bib. Canonical variable 2 primarily differentiates individuals based on the proportion of yellow, with high values indicating a high proportion of yellow, and low proportions of red and grey. Canonical variable 3 was associated with a low proportion of grey, high proportion of red, and high chromatic contrast of the throat. The correct assignment rate for morphs in cross-validation was 86% for yellow, 70% for orange, 100% for grey, and 73% for orange+yellow.

**Fig 4:**
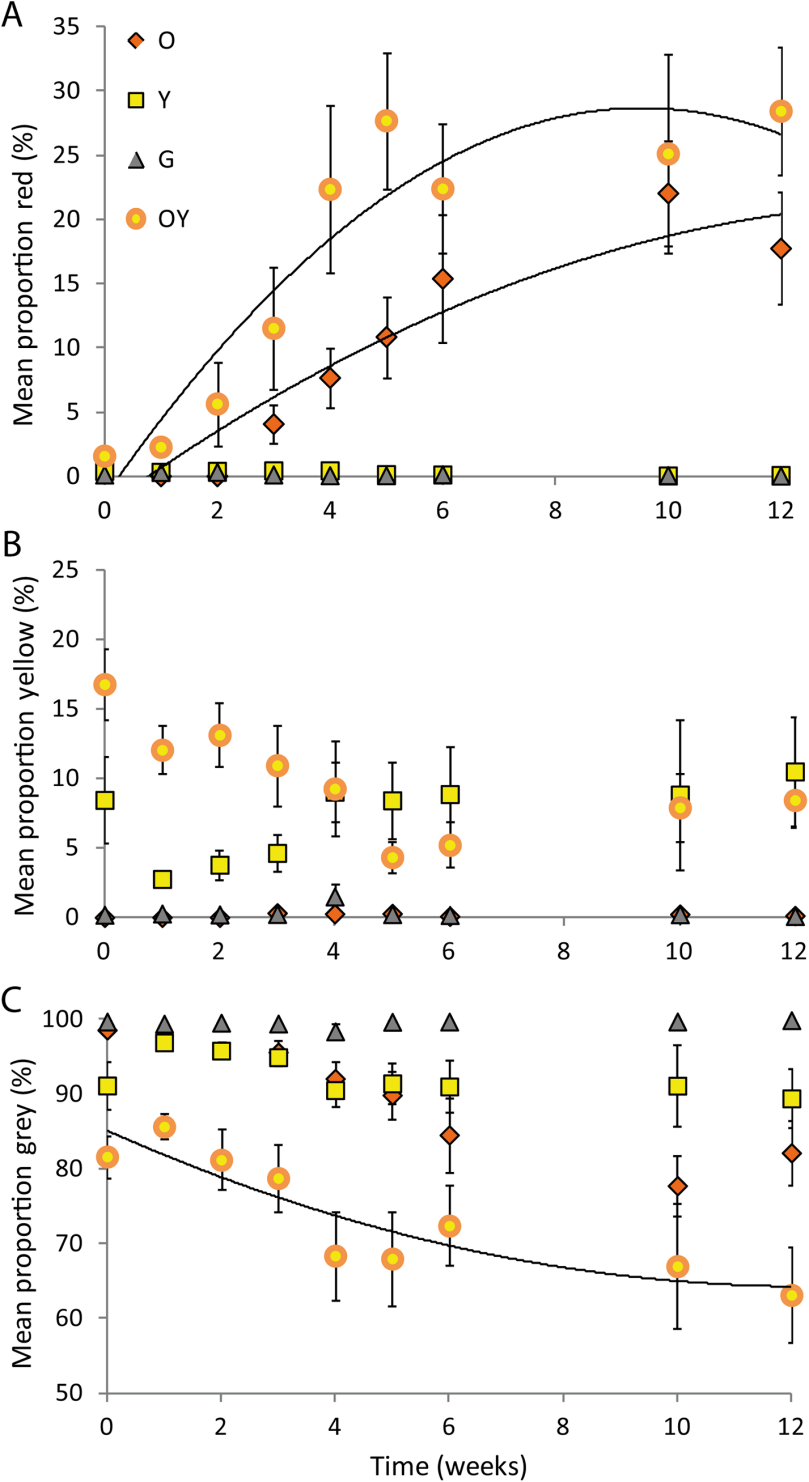
Mean percent of A) red, B) yellow and C) grey components of throat colouration over time. Data for each morph category were derived from digital images using segmentation analysis (n = 40). Significant effect of time for proportion red in O and OY morph, and proportion grey in OY morph (quadratic trendline fitted); p < 0.0001 in each case.

Analysis of the change in the proportion of red, yellow and grey (from segmentation analysis of weekly photographs) over time showed strong differences between morphs (Table 1). There was a significant interaction between colour morph and time for both the proportion of red and proportion of grey (Table 1). There was a marked, quadratic increase in the proportion of red and decrease in grey over time for the orange and orange+yellow morphs; while there was no change for the yellow or grey morphs (Fig 4A and 4C; Table 2). For the proportion of yellow, there was no significant interaction between morph and time (Table 1), and no consistent increase or decrease in the proportion of yellow through time (Fig 4B). However, there was a significant difference in the proportion of yellow between morphs (Table 1) with the yellow and orange+yellow morphs having a higher proportion of yellow than the orange or grey morphs (yellow vs grey t_246_ = 5.01, p < 0.0001; yellow vs orange t_246_ = 5.48, p < 0.0001; orange+yellow vs grey t_246_ = 4.02, p < 0.0001; orange+yellow vs orange t_246_ = 4.50, p < 0.0001; Fig 4C).

**Table 1:** Effect of testosterone implantation on components of female colour expression. The change in proportion of throat colours (red, yellow and grey) was derived from segmentation analysis of digital images, and the chromatic (colour) and achromatic (luminance) contrast of the primary throat and bib was derived from spectral measurements. For significant quadratic interactions between morph and time (denoted by *), each morph was analysed separately (Table 2). Values remaining significant after applying FDR corrections in bold.

Dependent	Fixed factor	F_df_	p-value
**Proportion Red**	Time	56.34_1,270_	**<0.0001**
	Morph	1.04_3,270_	0.3746
	Morph x Time	29.36_3,270_	**<0.0001**
	Time^2^	29.21_1,270_	**<0.0001**
	Morph x Time^2^	14.42_3,270_	<0.0001*
**Proportion Yellow**	Time	1.86_1,270_	0.1742
	Morph	10.60_3,270_	**<0.0001**
	Morph x Time	0.40_3,270_	0.7544
	Time^2^	0.80_1,270_	0.3721
	Morph x Time^2^	0.35_3,270_	0.7864
**Proportion Grey**	Time	51.87_1,269_	**<0.0001**
	Morph	6.08_3,269_	0.0005
	Morph x Time	16.55_3,269_	**<0.0001**
	Time^2^	23.34_1,269_	**<0.0001**
	Morph x Time^2^	6.91_3,269_	0.0002*
**Chromatic Contrast: Throat**	Time	125.87_1,270_	**<0.0001**
	Morph	1.25_3,270_	0.2909
	Morph x Time	28.79_3,270_	**<0.0001**
	Time^2^	64.49_1,270_	**<0.0001**
	Morph x Time^2^	15.07_3,270_	<0.0001*
**Achromatic Contrast: Throat**	Time	203.45_1,270_	**<0.0001**
	Morph	0.62_3,270_	0.6034
	Morph x Time	0.99_3,270_	0.3985
	Time^2^	130.23_1,270_	**<0.0001**
	Morph x Time^2^	0.68_3,270_	0.5668
**Chromatic Contrast: Bib**	Time	2.06_1,242_	0.1523
	Morph	4.56_3,242_	**0.0040**
	Morph x Time	0.58_3,242_	0.6290
	Time^2^	9.07_1,242_	**0.0029**
	Morph x Time^2^	0.81_3,242_	0.4891
**Achromatic Contrast: Bib**	Time	52.49_1,242_	**<0.0001**
	Morph	0.80_3,242_	0.4941
	Morph x Time	0.18_3,242_	0.9079
	Time^2^	45.73_1,242_	**<0.0001**
	Morph x Time^2^	0.60_3,242_	0.6174

**Table 2:** Linear and quadratic changes in colour over time for each morph separately. Only colour variables for which there was a significant interaction between colour morph and time are presented. Values remaining significant after applying FDR corrections in bold.

Dependent	Morph	Fixed factor	F_df_	p-value
**Proportion Red**	Grey	Time	1.95_1,54_	0.1681
		Time^2^	0.03_1,54_	0.8612
	Orange	Time	31.78_1,76_	**<0.0001**
		Time^2^	11.19_1,76_	**0.0013**
	Orange-Yellow	Time	72.85_1,86_	**<0.0001**
		Time^2^	39.90_1,86_	**<0.0001**
	Yellow	Time	7.34_1,54_	**0.0090**
		Time^2^	0.63_1,54_	0.4314
**Proportion Grey**	Grey	Time	0.06_1,54_	0.7999
		Time^2^	2.15_1,54_	0.1479
	Orange	Time	31.05_1,76_	**<0.0001**
		Time^2^	8.96_1,76_	**0.0037**
	Orange-Yellow	Time	60.98_1,85_	**<0.0001**
		Time^2^	29.64_1,85_	**<0.0001**
	Yellow	Time	0.21_1,54_	0.6483
		Time^2^	0.27_1,54_	0.6033
**Chromatic Contrast: Throat**	Grey	Time	0.87_1,54_	0.3563
		Time^2^	0.15_1,54_	0.7047
	Orange	Time	66.33_1,76_	**<0.0001**
		Time^2^	33.02_1,76_	**<0.0001**
	Orange-Yellow	Time	116.28_1,86_	**<0.0001**
		Time^2^	60.31_1,86_	**<0.0001**
	Yellow	Time	6.43_1,54_	**0.0142**
		Time^2^	4.01_1,54_	0.0503

**Fig 5:**
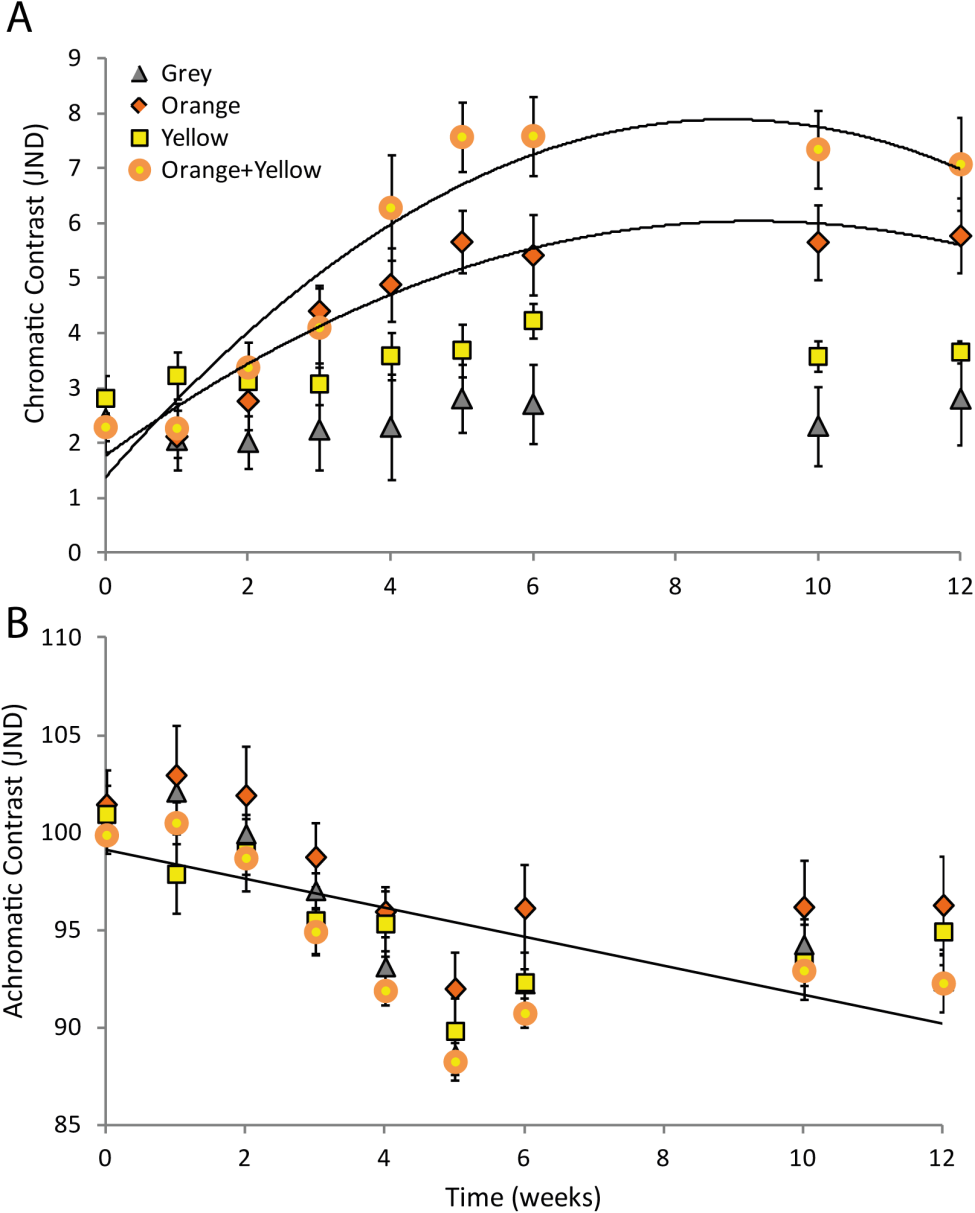
A) Chromatic (CC; colour) and B) achromatic (AC; luminance) contrast of primary female throat colouration against a neutral background (30% grey), for each colour morph. CC and AC measured in units of just noticeable differences (JND).

There was a significant interaction between colour morph and time for the chromatic (colour) contrast of the primary throat colour (Table 1). The chromatic contrast increased over time for the orange and orange+yellow morphs; while there was no consistent change for the grey or yellow morphs (Fig 5A; Table 2). There was no significant interaction between colour morph and time for the achromatic contrast of the throat, nor the chromatic or achromatic contrast of the bib (Table 1). Achromatic contrast of the throat and bib decreased over time in all morphs: the throat reduced overall brightness as it changed from cream to coloured over time, irrespective of morph (Fig 5B). Chromatic contrast of the bib decreased moderately over time but was consistently higher in the yellow and orange+ yellow morphs than the orange or grey morphs (yellow vs grey t_218_ = 3.01, p = 0.003; yellow vs orange t_218_ = -2.87, p = 0.0045; orange+yellow vs grey t_218_ = -3.50, p = 0.0006; orange+yellow vs orange t_246_ = -3.43, p = 0.0007).

## Discussion

Our results demonstrate that testosterone manipulation in *C*. *decresii* induces females to express the same set of morph classes as males (orange, yellow, grey and orange+yellow) and in similar relative frequencies. We found that the colour morphs in females can be classified with a similar degree of confidence as in males [27]. Although cross-validation tests showed moderate misassignment frequencies for the orange and orange+yellow morphs, this is mainly due to the stringent segmentation threshold for the proportion of red on the throat; the orange and orange+yellow morphs are easily visually distinguished. However, we observed that testosterone does not influence yellow colour expression in the same way as orange and grey. While the proportion of orange and chromatic contrast of the throat increased dramatically in the orange and orange+yellow morph due to development of a central orange patch, there was little change in the size or intensity of the yellow component of the throat. As such, the orange+yellow morph in females does not have the same characteristic central orange patch surrounded by yellow as males, nor does the yellow colouration of yellow morphs extend across the whole throat. Rather, the yellow in yellow and orange+yellow females is largely restricted to the bib and lower throat.

In species with sex-limited colour polymorphism, the phenotypic diversity (i.e. polymorphism) is only observable in one sex (usually males), so it is difficult to assess the contribution of the female genome; the unexpressed alleles she will pass to her sons. Hormone manipulation, specifically testosterone administration, has previously been used to induce polymorphic colouration in female ruffs, *Philomachus pugnax* [21] and painted dragons, *Ctenophorus pictus* [14]—a close relative of *C*. *decresii*. Here, we have demonstrated that it is similarly possible to induce female *C*. *decresii* to express the alleles they possess for throat colouration, which contributes to our understanding of the underlying patterns of inheritance in this species. In the majority of polymorphic species for which we know the genetic mode of inheritance, colour expression is controlled by a single autosomal locus or super-gene and shows Mendelian patterns of inheritance [7, 38, 39]. Agamid lizards have a ZZ/ ZW sex determination system (with temperature dependent sex determination), with females the heterogametic sex [40, 41]. As both sexes have a Z chromosome, we cannot eliminate the possibility that in *C*. *decresii*, genes coding for throat colour directly (or for modifiers that affect expression indirectly) are sex-linked if there is dosage compensation. However, the expression of female colouration in response to the male sex steroid testosterone indicates that the colour polymorphism is most likely controlled by one or more autosomal loci.

Testosterone influences cellular activity in target tissues [42]; however, the specific mechanisms linking plasma testosterone concentrations and pigment synthesis are poorly understood [43]. In female *C*. *decresii*, testosterone implantation influenced the expression (both proportion and intensity) of orange much more strongly than yellow. In reptiles, yellow–red colouration is generated by carotenoids, pteridines, or a combination of these two classes of pigment [26, 35, 44]. In *C*. *decresii*, preliminary assays suggest that yellow and orange throat colouration is primarily generated by pteridine pigments [Pryke and Stuart-Fox, unpublished data], which are synthesised within pigment cells from common precursors [26, 44, 45]. Testosterone has been suggested to be involved in pteridine biosynthesis [46, 47], so the production of yellow may involve different precursors and/ or cofactors than the production of orange [20]. By contrast, there is little evidence that testosterone affects the expression of carotenoid-based orange colouration in lizards [20]. The disparity, therefore, between expression of yellow and orange may indicate differential effects of testosterone on different pigment types underlying the colour expression. Additionally, the expression of orange and yellow colouration could potentially involve different genetic or developmental mechanisms and different associated proximate and ultimate costs. The proportion of orange-throated males increases and yellow-throated males decreases with increasing aridity among populations of *C*. *decresii* [McLean, Stuart-Fox & Moussalli, unpublished data]. Furthermore, in *C*. *decresii*, orange throat colouration is more conspicuous than yellow against the rock and lichen background of their native habitats [3]. Thus, it is plausible that there are different ultimate costs of yellow versus orange colour expression in this species, which may explain why mature females express some yellow but not orange throat colouration. Further research is needed to better understand how testosterone influences expression of specific pigment classes and the proximate and ultimate costs of expression of yellow versus orange colouration.

Our results are concordant with a number of both correlative and manipulative studies showing that testosterone induces female colour expression in lizards. For example, development of orange colouration on the throat or ventro-laterally is associated with elevated circulating plasma testosterone levels in female spiny lizards, *Sceloporus pyrocephalus* [25], both captive and wild female-keeled earless lizards, *Holbrookia propinqua* [48] and captive Lake Eyre dragon lizards, *Ctenophorus maculatus* [49]. Female colour expression is also intensified by experimental elevation of testosterone using implants, such as in eastern fence lizards, *Sceloporus undulatus* [13, 50]. Interestingly, in all these cases, testosterone is associated with expression of orange colouration in females, although it is not known whether orange is generated by carotenoid or pteridine pigments in these species. In *C*. *decresii*, a number of females exhibited some degree of yellow bib and lower throat colouration prior to testosterone implantation whereas orange colouration was entirely absent. We observed dramatic increases in the proportion and intensity of orange over time but no similar increase in yellow. Thus it is possible that yellow and orange+yellow morph females express alleles for yellow colouration from sexual maturity, whereas alleles for orange colouration are not expressed. An analogous situation may occur in side-blotched lizards, *Uta stansburiana*, where males are polymorphic with blue, orange or yellow throats (carrying *b*, *o* or *y* alleles respectively) whereas females express minor amounts of blue or orange but no yellow such that *oy* versus *oo* and *by* versus *bb* phenotypes cannot be distinguished [51].

The effects of testosterone on colour expression may depend on the developmental stage at which it is applied [15, 52], and may continue beyond the period over which plasma testosterone levels are elevated [53]. In our study, circulating testosterone levels at four weeks were still elevated relative to pre-treatment samples but had dropped significantly from one-week after implantation. We monitored colour expression over 12 weeks but do not know whether testosterone remained elevated beyond four weeks. While across our treatment population there is little evidence of colour reversion, several individual lizards lost their throat colouration after their peak in expression (ranging from three to twelve weeks). Salvador, Veiga [20] speculate that in the lizard *Psammodromus algirus*, the effect of testosterone is age dependent, given the possible molecular mechanisms that can influence hormone responses at different target tissues and life-history stages. There may be a developmental window (either prenatal or at adolescence) that will cause the induced colour to remain fixed [12, 15, 54, 55]. Thus, the large variation we observed in the extent and duration of colour expression among individual *C*. *decresii* females may partially be accounted for by variation in age, which ranged from 2 to >5 years.

Given the lack of a treatment with an empty implant, it is possible that the stress of the implant, rather than testosterone, affected colour expression, as glucocorticoids can modify the expression of pre-existing colouration in lizards [23, 56–58]. However, our results show that testosterone implantation led to the appearance of orange colouration (never naturally observed in females) in only a subset of females and did not significantly affect the expression of yellow. It seems unlikely that stress response hormones could induce the full expression of male colour polymorphism, and specifically orange, in this way. Thus, we are confident that the observed changes in colour expression reflect the effects of testosterone rather than the stress of implantation *per se*.

Overall, we have demonstrated that females in the polymorphic lizard *C*. *decresii* express the same set of colour morphs as males and in similar relative morph frequencies. However, the effect of testosterone differs for orange and yellow, suggesting that their expression may be governed by different proximate mechanisms. This has important implications for understanding the relative costs and information conveyed by these two colour components of the throat signal. Furthermore, differential induction of orange and yellow by testosterone suggests that caution is required when using testosterone implantation in females to study the genetic basis of colour polymorphisms in lizards.

## Supporting Information

S1 Dataset Raw data. (XLSX)

S1 Fig Reflectance of grey, yellow and orange across 300–700nm; the visual spectrum of lizards. Colours have minimal ultraviolet reflectance (300–400nm).
(PDF)
