# Supplementary material for: Testosterone-Induced Expression of Male Colour Morphs in Females of the Polymorphic Tawny Dragon Lizard, Ctenophorus decresii
Source: PLoS One. 2015 Oct 20;10(10):e0140458. doi: 10.1371/journal.pone.0140458 (PMC4615632; doi:10.1371/journal.pone.0140458)
Supplement: S1 Fig — Colours have minimal ultraviolet reflectance (300–400nm). (PDF) [file pone.0140458.s002.pdf]

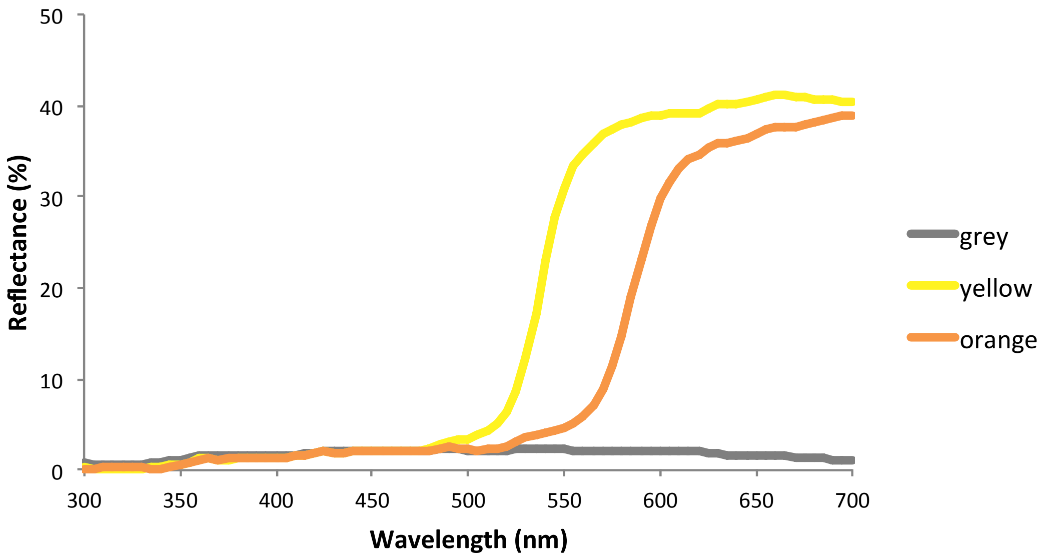

Fig. S1. Reflectance of grey, yellow and orange across 300 - 700nm; the visual spectrum of lizards. Colours have minimal ultraviolet reflectance (300 - 400nm)
